# Storage and Algal Association of Bacteria That Protect *Microchloropsis salina* from Grazing by *Brachionus plicatilis*

**DOI:** 10.3390/microorganisms11030786

**Published:** 2023-03-18

**Authors:** Carolyn L. Fisher, Michelle V. Fong, Pamela D. Lane, Skylar Carlson, Todd W. Lane

**Affiliations:** 1 Physical and Life Science Directorate, Nuclear and Chemical Sciences Division, Lawrence Livermore National Laboratory, Livermore, CA 94550, USA; 2 Department of Chemistry, University of the Pacific, Stockton, CA 95211, USA; 3 Systems Biology Department, Sandia National Laboratories, Livermore, CA 94550, USA; 4 Bioresource and Environmental Security Department, Sandia National Laboratories, Livermore, CA 94550, USA

**Keywords:** *Microchloropsis salina*, *Brachionus plicatilis*, bacterial communities, algal biofuels

## Abstract

Loss of algal production from the crashes of algal mass cultivation systems represents a significant barrier to the economic production of microalgal-based biofuels. Current strategies for crash prevention can be too costly to apply broadly as prophylaxis. Bacteria are ubiquitous in microalgal mass production cultures, however few studies investigate their role and possible significance in this particular environment. Previously, we demonstrated the success of selected protective bacterial communities to save *Microchloropsis salina* cultures from grazing by the rotifer *Brachionus plicatilis*. In the current study, these protective bacterial communities were further characterized by fractionation into rotifer-associated, algal-associated, and free-floating bacterial fractions. Small subunit ribosomal RNA amplicon sequencing was used to identify the bacterial genera present in each of the fractions. Here, we show that *Marinobacter*, *Ruegeria*, and *Boseongicola* in algae and rotifer fractions from rotifer-infected cultures likely play key roles in protecting algae from rotifers. Several other identified taxa likely play lesser roles in protective capability. The identification of bacterial community members demonstrating protective qualities will allow for the rational design of microbial communities grown in stable co-cultures with algal production strains in mass cultivation systems. Such a system would reduce the frequency of culture crashes and represent an essentially zero-cost form of algal crop protection.

## 1. Introduction

Although the production of biofuels from microalgae is perceived as having advantages over those derived from terrestrial crops [1], significant barriers remain for successful commercial production [2]. Most notable of these is the failure to achieve the sustained annualized rate of areal production necessary for economically viable fuel production. Such an issue is especially problematic due to the cost limitations associated with the production of a relatively low-value product such as biofuel. Open-pond systems are thought to be more economically viable than closed-system photobioreactors for production of algal biomass for these applications [3]. However, both types of algal mass culture systems are susceptible to biocontamination and culture crashes requiring further analysis [4].

A wide variety of pest species have been implicated in biocontamination-mediated pond crashes including a range of viruses, fungi, protozoans, and harmful bacteria [5]. A recent review [6] specifically argues that biocontamination is among the top priority challenges still facing industrial scale production of microalgae and cyanobacteria for biofuel production. Grazing zooplankton, such as rotifers [7], are voracious algal consumers and thus rank highest among the most destructive grazers inducing catastrophic pond crashes [8]. The grazer *Brachionus plicatilis*, a common contaminant in marine algal ponds, is capable of ingesting ~200 microalgal cells per minute [9], or about 1200 to as many as 4800 algal cells per hour per individual [7], and has been the topic of a number of studies on methods of biocontamination control [8,10]. The freshwater rotifer *Brachionus calciflorus* similarly consumes 500 cells of algal cells per hour [11]. Mitigating the devastating impacts of zooplankton contamination on open algal production pond systems remains a primary challenge for algal industry for biofuel production and economy.

Previous studies have demonstrated the use of specific bacteria or bacterial consortia in co-culture with algae to inhibit pests from causing algal pond crashes [4,12,13]. In fact, violacein-producing bacteria can eradicate rotifers and other pest species within two days of co-cultivation [14], and more recently, Ward et al. demonstrated that the addition of the violacein-producing bacterium *Janthinobacter lividum* offers protection for *Microchloropsis salina* [15]. In both cases, protection from grazing required separate cultivation and introduction of the violacein–producing bacteria to the algal production system. *J. lividum* persisted in algal cultures for only three days, post inoculation in outdoor cultivation trials following addition and thus may not be suitable for long term prophylaxis and against microalgal grazers. Furthermore, the costs associated with separate cultivation and addition of violacein–producing bacteria may limit their application to the production of low-value products, not as a protective microalgal community member.

In previous work [12], we demonstrated the selection of a bacterial assemblage capable of protecting cultures of *M. salina* from grazing-induced crashes by *B. plicatilis*. We demonstrated that this assemblage could persist for long periods of time in stable co-culture with *M. salina* and continue to provide protection against rotifer grazing which abrogates the requirement for and expense of separate cultivation of protective bacteria. Through SSUrRNA amplicon sequencing, we identified bacterial genera that were positively correlated with protective communities: one unclassified Rhodobacteraceae genus, two *Marinobacter* sp., two *Pseudomonas* sp., one *Ruegeria* sp., and one *Paracoccus* sp.

In this study, we identify bacterial community members that will live commensally within microalgal cultures and provide protective benefits, preventing infestation by algal parasites, grazers, and other pests. We expand upon our prior work on these bacterial assemblages by subjecting the samples to size fractionation via sequential filtration, followed by community analysis by SSUrRNA amplicon sequencing. Such testing enables this study to determine whether protective members of the bacterial assemblage were differentially associated with the algae or rotifers fractions or in the bulk fraction. These results suggest new opportunities for how to best cultivate microbial assemblages alongside microalgae for biofuel production systems.

## 2. Materials and Methods

### 2.1. Algal, Rotifer, and Algal–Bacterial Cultures

Axenic cultures of *Microchloropsis salina* CCMP 1776 were obtained from the NCMA (Bigelow Laboratory, Boothbay Harbor, ME, USA). Axenicity was not further verified. Cultures of *M. salina* alone and *M. salina* in the presence of seven bacterial communities identified in Fisher 2019 were grown, separately, under the same conditions previously described, including modified ESAW medium [16]. Batches of 20,000 cysts for L-type marine rotifer *Brachionus plicatilis* (Florida Aqua Farms, Dade City, FL, USA) were sterilized as described in [12] and hatched in sterile ESAW at 28 °C for 48 h. Axenic rotifer cultures were maintained at approximately 22 °C in 1 L of sterile ESAW medium and fed ~300 M cells of axenic *M. salina* every other day. Rotifer density was determined by light microscopy counts.

Bacterial communities were generated, cultured, and maintained as described previously [12]. Freezer stocks were previously prepared from these original algal–bacterial co-cultures and stored in 25% DMSO. For this study, cultures from cryovials with two different dates were selected for this study and labeled as “_.1” and “_.2”. In this way, the “algal–bacterial co-culture” of 2p [12] resulted in the descendants of 2p.1 and 2p.2, and so on for the other bacterial communities in this study. Bacterial communities were cultured with 10–15 M mL^−1^ of axenic *M. salina*, maintained with weekly ESAW dilutions (1:10), and challenged with 10–20 rotifers mL^−1^, [12] for four weeks before being assayed (detailed below).

### 2.2. Microalgal Growth Assay, Specific Growth Rate Calculation, and Rotifer Counts

**Figure 1:**
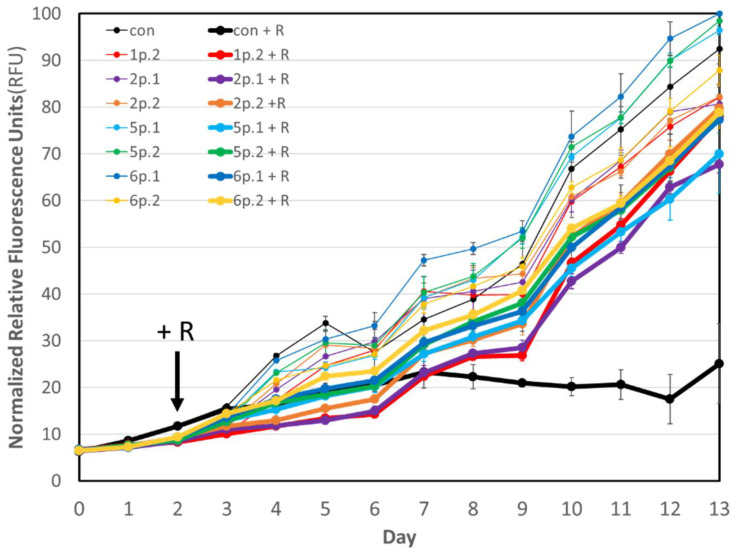
Daily chlorophyll fluorescence for *M. salina* cultures treated with bacterial communities (1p.2, 2p.1, 2p.2, 5p.1, 5p.2, 6p.1, 6p.2) in comparison to an untreated *M. salina* control (con) in the presence of rotifers (+R; thick lines) and absence (thin lines). Measurements were averaged for biological triplicates for each condition and error bars represent standard deviation (*n* = 3). Rotifers were added on day 2. Data were normalized to the culture with the highest chlorophyll fluorescence (6p.1) over the cultivation period.

*M. salina* algal cultures were cultured in the presence of bacterial communities (1p.2, 2p.1, 2p.2, 5p.1, 5p.2, 6p.1, and 6p.2). Algal growth and rotifer grazing assays were carried out in triplicate, as in [12]. Briefly, a total of 48 culture flasks, each containing 25 mL of ESAW medium, and ~1 M mL^−1^ *M. salina*, were used. We divided the 48 cultures into 8 groups of 6 replicate flasks: seven sets were each treated with one of the 7 bacterial communities, while the 8th was left as an untreated control. After 48 h of growth, 10 axenic *B. plicatilis* rotifers per mL were added to three of the six replicates for each set of conditions. To measure algal density and growth, daily timepoint chlorophyll fluorescence readings (430 nm excitation, 685 nm emission) were taken as 200-uL subsamples, in duplicate, from the 48 individual flasks at days 0–13 and measured with a Tecan i-control infinite 200Pro version 1.11.1.0. Fluorescence data were normalized to the sample with highest relative fluorescence (day 13 for 6p.1 without rotifers), as shown in Figure 1. Table 1 summarizes normalized, average specific growth rates calculated for each culture condition and rotifer counts made by light microscopy on days 6, 11, and 13. As previously described [12], specific algal growth rates (day^−1^) were calculated 24 h after addition of rotifers (for days 3–13) as the slope over the natural log of chlorophyll fluorescence. These values were averaged (*n* = 3) and then normalized to the untreated *M. salina* control (without rotifers) for each condition. Rotifer counts were determined using light microscopy.

### 2.3. Fractionation by Sequential Filtration

To characterize the association of protective bacteria in treated *M. salina* cultures in the presence and absence of rotifers, day 13 cultures were sequentially filtered through a 41 µm pore-size nylon cell strainer follow by two different pore-sized polyethersulfone filters (ThermoFisher Scientific, MA): the 41 µm retentate (41 ret) was used to collect rotifers and associated bacteria, the 0.8 µm retentate (0.8 ret) was used to collect algae and associated bacteria, and 0.22 µm retentate (0.22 ret) was used to collect all other free-living bacteria. After each filtration step, retained biomass was washed from the filter using 0.8 mL ESAW medium, collected, and pelleted by centrifugation at 15,000 RPM for 20 min. Cell pellets were stored at −80 °C prior to DNA extraction and sequencing analysis.

### 2.4. DNA Extraction, Library Preparation, SSUrRNA Amplicon Sequencing, and Data Analysis

**Figure 2:**
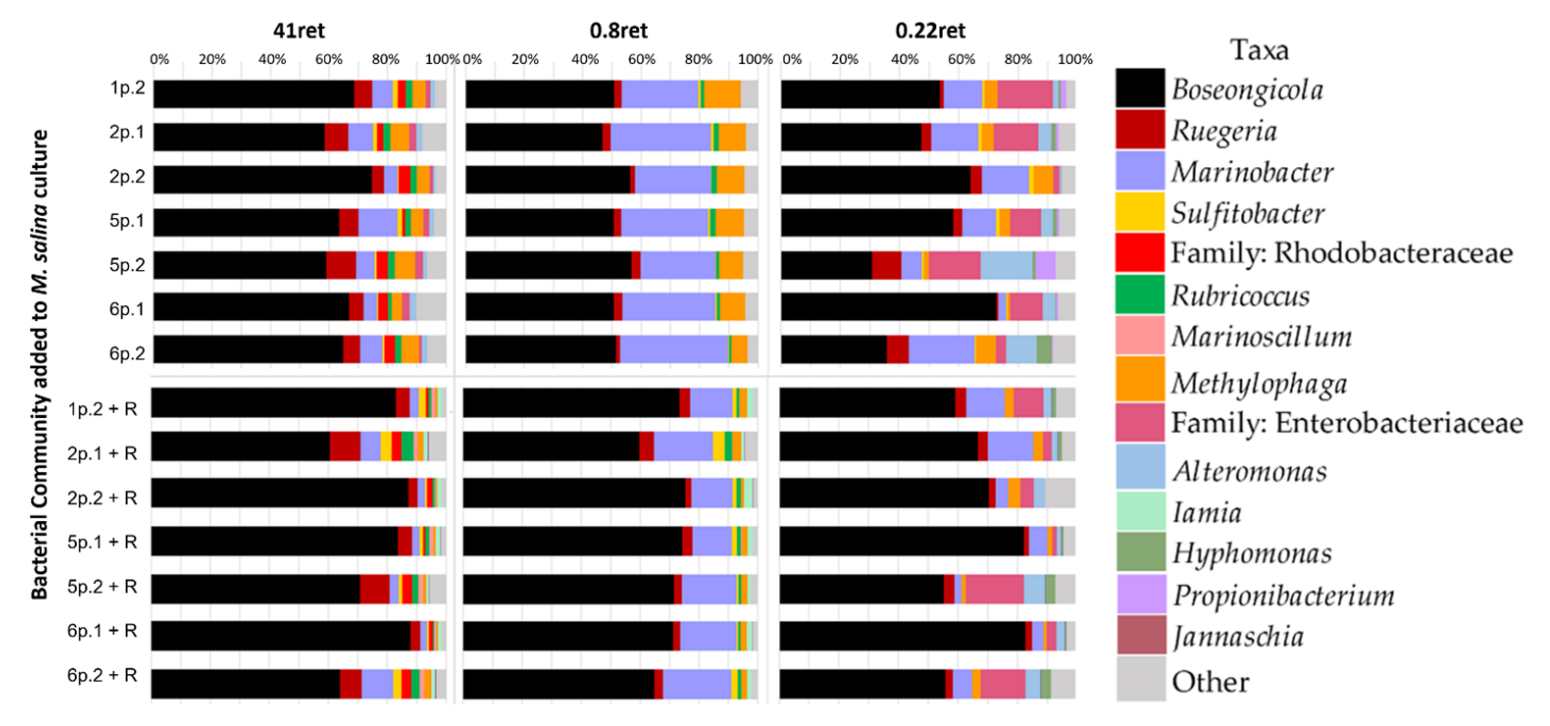
Averaged stacked bar graph of the bacterial OTUs present in different fractions in the absence (top) and presence (+R, bottom) of *B. plicatilis*. Taxonomy is shown at the genus level unless otherwise stated. Bacterial OTUs <1% of the total abundance is categorized in ‘Other’ (grey).

Samples were prepared for SSUrRNA gene amplicon sequencing [12] using Illumina MiSeq (Illumina, San Diego, CA, USA) to perform paired-end 300-bp sequencing at Sandia National Laboratories. Sequencing read library analyses were performed with QIIME 2 2019.7.0 [17] using default settings for quality-filtering and de-multiplexing sequences. All genus-level orthogonal taxonomic units (OTUs) were determined using the reference database Greengenes version v13.5. Algal and mitochondrial DNA sequences were filtered and removed from bacterial community data. Bacterial OTUs were totaled across all samples for each biological condition (n = 3) and sorted according to total relative abundance of all samples. Total OTUs were then calculated from the totals of each row. Percent relative abundance was calculated for each OTU using this total and individual row totals to sort bacteria between present in >1% and <1% of all samples. For OTUs present in >1% of all samples, the percent relative abundance was calculated for 41 ret, 0.8 ret, and 0.22 ret fractions and these taxa are shown in Figure 2. For OTUs present in <1% of all samples, taxa were aggregated to an “Other <1%” category in Figure 2. 

## 3. Results

### 3.1. Microalgae Growth Experiment

The present study confirms our previous results [12] that indicated that presence of the protective consortia prevented grazing by rotifers and resulted in a decrease in rotifer number. Samples of the original algal–bacterial co-cultures (1p, 2p, 5p, and 6p) were shown to be protective against rotifer grazing [12] and then cryogenically frozen and stored for four months. The descendent bacterial communities (1p.2, 2p.1, 2p.2, 5p.1, 5p.2, 6p.1) re-established from these frozen stocks were assayed for their protective potential in the same microalgal growth assay and were shown to prevent complete algal loss by rotifer grazing in comparison to untreated *M. salina* in the presence of active rotifers (Table 1 and Figure 1). Thus, it is evident that cryogenic storage (approximately 4 months) did not adversely impact the protective capability of the seven assayed bacterial communities. Addition of protective bacteria to *M. salina* did not inhibit microalgal growth in the absence of rotifers, and in some cases resulted in higher growth rates, such as for 5p.1, 5p.2, and 6p.1. 

### 3.2. Bacterial Community Composition

Upon completion of the microalgal growth assay, samples were sequentially filtered through three different pore-sized filters: 41 µm, to collect rotifers, flocculated algal cells, and associated bacteria; 0.8 µm to collect algae and associated bacteria; and 0.22 µm to collect all other bacteria. The three retentates collected are referred to henceforth as “41 ret”, “0.8 ret”, and “0.22 ret”, respectively. Eubacterial OTU community members were identified to the family or genus level. Relative abundances, shown in Figure 2, are the average of the three biological replicates (values for individual biological replicates are shown in Online Resource 1, Figures S1–S3). All seven bacterial communities showed similarities between rotifer-associated (+R) and rotifer-free (-R) cultures. Specifically, the genera *Boseongicola*, *Marinobacter*, *Methylophaga*, and *Ruegeria* were present, at various relative abundances, in all +R and -R fractions. Unlike the original bacterial communities, *Pseudomonas* and *Paracoccus* were not found in high relative abundance in the re-established cultures. Although members of these genera were correlated with protection in the parental cultures, they are, at least, not required in high abundance for these seven bacterial communities to be protective. 

Sequential filtration allowed for the determination of whether specific protective community members were enriched in specific fractions suggesting their association with the algae or rotifers. *Marinobacter* sp. was present in every sample sequenced, at varying relative abundances between 5–30%, in this study (Figure 2). However, the 0.8 ret fraction, containing algae, was enriched in *Marinobacter* for both +R and -R communities compared to the 41 ret and 0.22 ret fractions. The relative abundance of *Marinobacter* in the 0.8 ret fraction of the -R cultures ranged from at 22% to 40% while for +R cultures it ranged from 15% to 25%. Besides *Marinobacter*, other notable differences between +R and -R bacterial communities include a higher relative abundance of Sulfitobacter in +R cultures and of *Methylophaga* in -R cultures.

For this present study, each algal–bacterial community sample tested had between 30 and 90% relative abundance of one recently identified Rhodobacteraceae family member, *Boseongicola* sp. (Figure 2). *Boseongicola* was not identified previously [12], but may be one of the unclassified Rhotobacteriaceae identified. This difference in identification is likely due to the difference in the databases used in these two works. Interestingly, there is one other unclassified Rhodobacteriaceae family member that was only correlated with the 41 ret samples in both -R and +R (Figure 2). These different correlations, with *Boseongicola* present in all samples at varying levels and the unclassified Rhodobacteriaceae family member present only in 41 ret samples, suggests different ecological roles for each bacterium. This was similarly concluded with the two Rhodobacteriaceae members identified previously [12].

*Ruegeria* is also member of the Rhodobacteriaceae family and was also found at varying relative abundances in almost every sample sequenced in this study (Figure 2). Although the pathogenicity of *Ruegeria* toward marine rotifers has not been determined, the *Ruegeria* sp. R11 has been shown to be an opportunistic pathogen of the ubiquitous *Emiliania huxleyi* [18]. Additionally, *Ruegeria mobilis* has been found to have probiotic potential for fish aquaculture due primarily to the production of tropodithietic acid as a potent antibacterial for fish pathogens [19]. Similarly, it is possible that the *Ruegeria* sp. present in the bacterial communities in this study might similarly produce chemicals that are antagonistic to marine rotifers in the culture.

*Alteromonas* was found in 0.22 ret samples for +R and -R samples and was also observed in the 41 ret -R fractions. Previously, it has been found that *Alteromonas* sp. added to cultures of the green alga *Dunaliela* sp. has been shown to aid in nitrogen uptake and increased biomass production [20]. Similarly, twelve strains of *Alteromonas macleodii* have been investigated and found to have various ecological roles to support microbial interactions in their various environments, such as secretion of amino acids, degradation of phenol and toluene, and production of siderophores and homoserine lactones [21]. Since *Alteromonas* was observed to be thriving in the *M. salina* cultures in this study, it is possibly assuming one of these roles.

The fractionation performed in this study resulted in a higher-resolution view of the bacterial community members. *Iamia* was also more specifically associated with +R bacterial communities in the 41 ret and 0.8 ret fractions. *Hyphomonas* was also exclusively found in the 0.22 ret fraction for both +R and -R communities. Interestingly, an unclassified Enterobacteraceae family member was observed predominantly in the 0.22 ret fractions for both -R and +R samples. It is possible that genera not observed in the previous study [12] could be due to their relatively lower abundance in comparison to other genera and the lack of fractionation performed in that analysis. 

## 4. Discussion

In Fisher et al. 2019, microbiome analyses of the original protective bacterial communities (prior to cryopreservation and storage) showed that the presence of *Pseudomonas*, *Ruegeria*, two *Marinobacter* species, and two unclassified Rhodobacteraceae genera were positively correlated with algal protection from rotifer grazing and found in all protective bacterial communities. *Marinobacter* have been shown to produce and release siderophores [22] which has been found to be detrimental to predators while simultaneously enhancing algal production [23]. *Paracoccus* and *Alteromonas* were correlated with protection in a subset of protective consortia. Overall, the majority of the identified bacteria consisted of the Proteobacteria phylum, and most were classified in the family Rhodobacteraceae. 

Based on the data presented herein, we show key taxa (*Marinobacter*, *Ruegeria*, *Boseongicola*) present in the added protective bacterial communities prevented grazing by *B. plicatilis*. This study shows the difference in bacterial composition of the bacterial communities in the presence and absence of *B. plicatilis* while showing specific bacteria associated with *M. salina*, *B. plicatilis*, and those unassociated. In this way, this study supports and extends the original study [12] and suggests possible mechanisms of community members observed to be specifically localized to algal or rotifer fractions. The demonstrated ability to cryopreserve and re-establish our protective consortia combined with their perseverance in co-culture with algae supports their utility as a cost-effective bio-control method to mitigate contamination by rotifers of microalgae for biofuel production.

## Figures and Tables

**Table 1:** Normalized average specific growth rate (day^−1^) of untreated *M. salina* (CON) or *M. salina* treated with bacterial communities (1p.2, 2p.1, 2p.2, 5p.1, 5p.2, 6p.1, 6p.2; described further in Section 2.1) and average motile rotifers (mL^−1^) present in cultures treated with rotifers (+R). All measurements were averaged for biological triplicates (*n* = 3) for each condition. Rotifers were added on Day 2.

Normalized Average Specific Growth Rate (Day^−1^)			Normalized Average Specific Growth Rate (Day^−1^)	Average Motile Rotifers mL^−1^		
				Day 6	Day 11	Day 13
**CON**	100	**CON + R**	13.5	25	75	37.5
**1p.2**	108.6	**1p.2 + R**	123.2	2.5	1.7	1.7
**2p.1**	117.0	**2p.1 + R**	105.8	10	5	0
**2p.2**	109.6	**2p.2 + R**	106.9	11.7	1.7	0.8
**5p.1**	115.2	**5p.1 + R**	133.6	22.5	5	8.3
**5p.2**	106.1	**5p.2 + R**	127.0	7.5	3.3	1.7
**6p.1**	115.0	**6p.1 + R**	112.8	15.8	9.2	0.8
**6p.2**	108.9	**6p.2 + R**	105.1	4.2	8.3	5.8

## Data Availability

Data is available upon request.

## Supplementary Materials

The following are available online at https://www.mdpi.com/article/10.3390/microorganisms11030786/s1, Figures S1–S3: Stacked bar graph of the bacterial OTUs consisting of 99% of the total abundance present in every individual culture representing bacteria associated with *B. plicatilis* (41 ret, Figure S1), associated with *M. salina* (0.8 ret, Figure S2), and unassociated bacteria (0.22 ret, Figure S3).
