# Supplementary material for: Storage and Algal Association of Bacteria That Protect Microchloropsis salina from Grazing by Brachionus plicatilis
Source: Microorganisms. 2023 Mar 18;11(3):786. doi: 10.3390/microorganisms11030786 (PMC10056100; doi:10.3390/microorganisms11030786)
Supplement: Supplementary file 1 [file microorganisms-11-00786-s001.zip › microorganisms-2259700-supplementary.pdf]

microorganisms MDPI

## Supplemental Figures

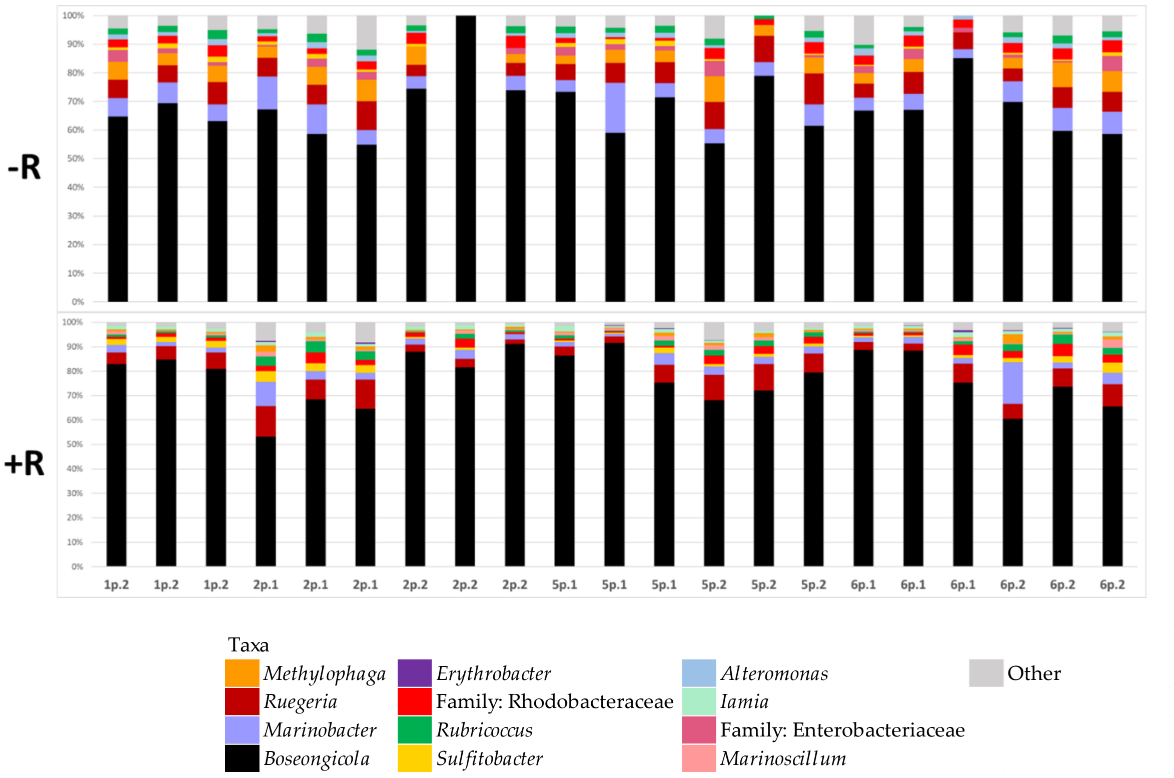


**Figure S1:** Stacked bar graph of the bacterial OTUs consisting of 99% of the total abundance present in every culture in the 40ret fraction. Bacterial OTUs shown represent bacteria associated with *B. plicatilis* in the absence (top, -R) and presence (bottom, +R) of *B. plicatilis*. Taxonomy is shown at the genus level unless otherwise stated. Bacterial OTUs less than 1% of the total abundance is categorized in 'Other' (grey).

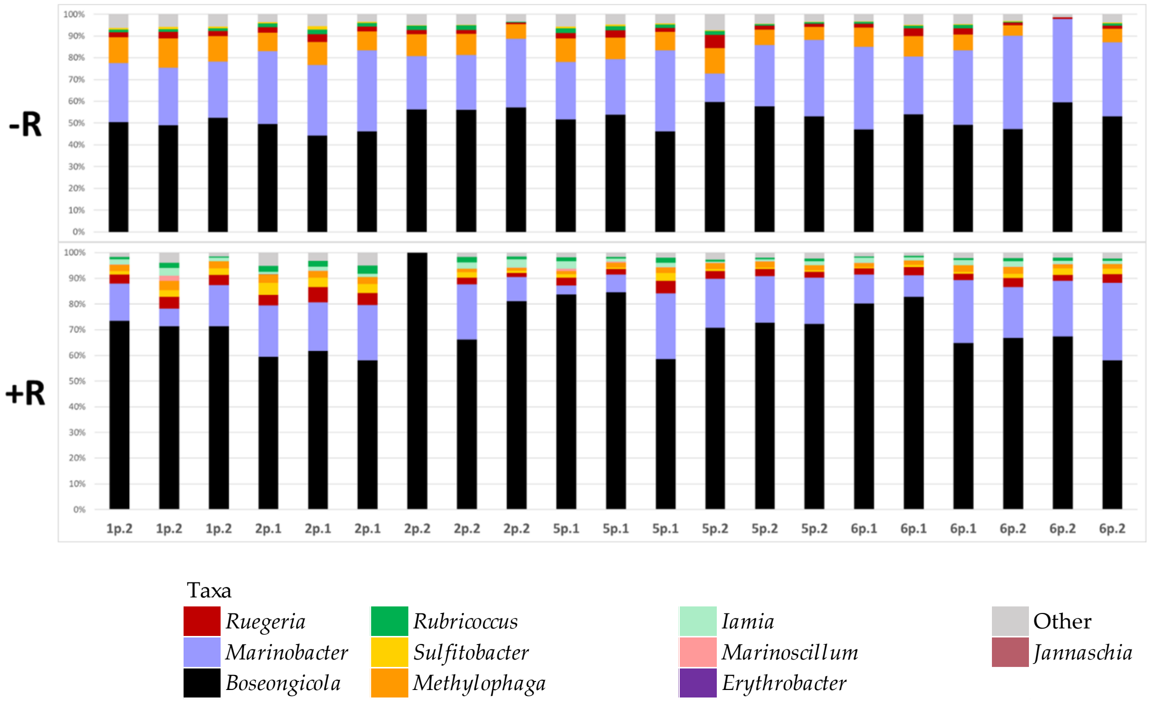


**Figure S2:** Stacked bar graph of the bacterial OTUs consisting of 99% of the total abundance present in every culture in the 0.8ret fraction. Bacterial OTUs shown represent bacteria associated with *B. plicatilis* in the absence (top, -R) and presence

(bottom, +R) of *B. plicatilis*. Taxonomy is shown at the genus level unless otherwise stated. Bacterial OTUs less than 1% of the total abundance is categorized in 'Other' (grey).

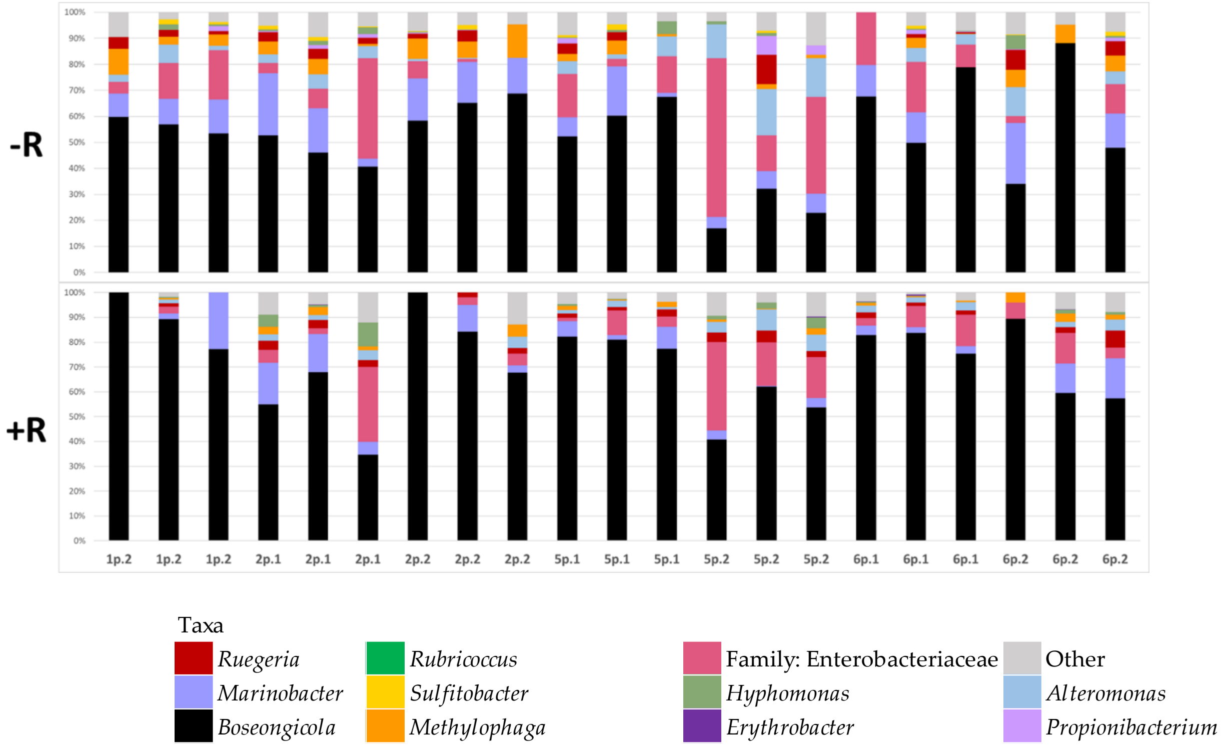


**Figure S3:** Stacked bar graph of the bacterial OTUs consisting of 99% of the total abundance present in every culture in the 0.22ret fraction. Bacterial OTUs shown represent bacteria associated with *B. plicatilis* in the absence (top, -R) and presence (bottom, +R) of *B. plicatilis*. Taxonomy is shown at the genus level unless otherwise stated. Bacterial OTUs less than 1% of the total abundance is categorized in 'Other' (grey).
